# Co-design of a school-based physical activity intervention for adolescent females in a disadvantaged community: insights from the Girls Active Project (GAP)

**DOI:** 10.1186/s12889-022-12635-w

**Published:** 2022-03-29

**Authors:** Sara McQuinn, Sarahjane Belton, Anthony Staines, Mary Rose Sweeney

**Affiliations:** 1 School of Nursing, Psychotherapy and Community Health, Faculty of Science and Health, Dublin City University, Dublin, 9 Ireland; 2 School of Health and Human Performance, Faculty of Science and Health, Dublin City University, Glasnevin, Dublin, 9 Ireland

**Keywords:** Physical activity, Behaviour change wheel, Adolescent female, Intervention development, School-based intervention, COM-B, TDF, BCTs

## Abstract

**Background:**

Globally, adolescents’ physical activity (PA) participation rates are low, particularly among lower socioeconomic groups, with females consistently the least active. The aim of this study was to co-design, with adolescent females, a school-based PA intervention in a single-sex, females-only designated disadvantaged post-primary school in Ireland. This involved using the Behaviour Change Wheel (BCW) and Public and Patient Involvement (PPI). This paper outlines the novel methodological approach taken.

**Methods:**

The three stages 1) understand the behaviour, 2) identify intervention options, and 3) identify content and implementation options of the BCW guide is described. A student PPI Youth Advisory Group (YAG) (*n* = 8, aged 15–17) was established. Mixed-methods were used with students (*n* = 287, aged 12–18) and teachers (*n* = 7) to capture current self-reported PA levels and to identify factors influencing adolescent females’ PA behaviour in their school setting. The intervention options, content and implementation options were identified through discussion groups with the YAG. The Template for Intervention Description and Replication (TIDieR) checklist was used to specify details of the intervention.

**Results:**

Just 1.4% of the students in this sample reported meeting the recommended PA guidelines. Students identified having more *‘time’* as the strongest predictor to becoming more active in school (Mean = 4.01, 95% CI 3.91 to 4.12). Social influences, environmental context and resources, behavioural regulation, beliefs about capabilities, goals, and reinforcement emerged from the qualitative data as factors influencing PA behaviour at school. The BCW co-design process resulted in the identification of seven intervention functions, four policy categories and 21 Behaviour Change Techniques. The Girls Active Project (GAP) intervention, a peer-led, after-school PA programme was proposed.

**Conclusions:**

This paper describes how the BCW, a comprehensive, evidence-based, theory-driven framework was used in combination with PPI to co-design a school-based intervention aimed to increase adolescent females’ PA levels. This approach could be replicated in other settings to develop targeted behavioural interventions in populations with specific demographic characteristics.

**Supplementary Information:**

The online version contains supplementary material available at 10.1186/s12889-022-12635-w.

## Background

Worldwide, more than three-quarters (81%) of adolescents (aged 11-17) do not meet the World Health Organisation recommended physical activity (PA) guidelines [1]. The World Health Organisation recommends that children and adolescents (aged 5–17) do at least an average of 60 min a day of moderate to vigorous physical activity (MVPA) [2]. The robust evidence that informed these guidelines on PA for children and adolescents found that greater amounts and higher intensities of PA are associated with multiple beneficial health outcomes, including cardiometabolic health, physical fitness, bone health, cognitive outcomes, reduced adiposity and a reduced risk of experiencing depression [3]. Research indicates that PA levels decline during adolescence [4–6] and that there are clear gender differences between the PA levels of adolescent females and males, with females less likely to meet recommended guidelines [1, 7, 8]. Evidence also suggests that socio-economic status is correlated to adolescents' PA participation [9, 10], where females with lower socio-economic status are consistently the least active [7]. Inactive adolescents are at a greater risk of long-term ill health [11], and given that participation in PA during adolescence can be a significant contributor to levels of PA in adulthood [12, 13], tackling the decline in PA during adolescence is a major public health priority.

There is no widely accepted explanation for the causes of adolescent females’ physical inactivity, but evidence suggest influences are multifactorial, including psychological, social and environmental factors [14, 15]. Females have cited many barriers to their participation, such as time restraints, competing priorities, social influences, low perceived competence, gender social stereotypes and a dislike for structured sports [16–21]. In addition, Martins et al. (2021) found that females and low socio-economic status adolescents faced even further difficulties with environmental factors that were perceived by adolescents to be barriers to PA, including space, infrastructure, equipment and unsuitable PA programmes [18]. Indeed, research indicates that targeting ecological domains beyond the individual level is important in PA interventions for adolescents [22], whilst tailoring these interventions to specific target populations is essential [23].

The current World Health Organisation ‘Global Action Plan on Physical Activity (2018–2030)’ stated the need to strengthen the development and implementation of behavioural public health interventions targeted at females and vulnerable or marginalised populations, that engage with and increase PA opportunities [24]. The school environment is well-known as a potential setting for targeting PA behaviour among adolescents [25, 26], yet the evidence for the effectiveness of school-based activity interventions is varied [27]. Love et al. (2019) found that current school-based efforts did not positively impact young people’s PA across the full day [28], whilst other reviews found multi-component interventions to be effective in the promotion of PA [29–31], with certain intervention strategies, such as after-school PA programmes indicating efficacy [30].

Public and Patient Involvement (PPI) is defined “as research being carried out ‘with’ or ‘by’ members of the public rather than ‘to’, ‘about’ or ‘for’ them” [32]. There is evidence to suggest that PPI in research may facilitate the process of translating research evidence into practice and policy [32, 33], contribute to reducing health research waste [34], and can have beneficial impacts for the service users, researchers and community [35], and on the quality and appropriateness of health research [36]. With the aim to capture the contextual input necessary to represent the unique youth experience, the use of PPI ‘youth advisory groups’ in health research has increased in recent years [37]. Of note, Dennehy et al. [38] found that young people can provide a unique perspective on the design, conduct and interpretation of research that it otherwise not accessible to adult researchers. Findings support however, that more work needs to be done to increase young people’s involvement in youth-related research [37, 39] and to enable them to influence the development of interventions that target health and well-being [40].

### Theoretical framework underpinning the intervention design

Best practice guidelines for developing interventions recommend using theory as a framework to design the intervention aimed at changing behaviour and suggest that complex interventions may work best if tailored to local circumstances [41]. Indeed, there is compelling evidence that theory-driven school-based PA interventions may be more effective in increasing adolescents females’ PA levels than non-theory based interventions [31, 42]. Michie et al. (2011) developed the Behaviour Change Wheel (BCW) from 19 behaviour change frameworks [43, 44]. It is a comprehensive method for developing interventions and is applicable to a range of health behaviours [44]. A ‘Behaviour Change Wheel Guide’ outlines three stages (eight steps) that should be used to design the behaviour change intervention [43]. These are described in the Methods section.

**Fig. 1:**
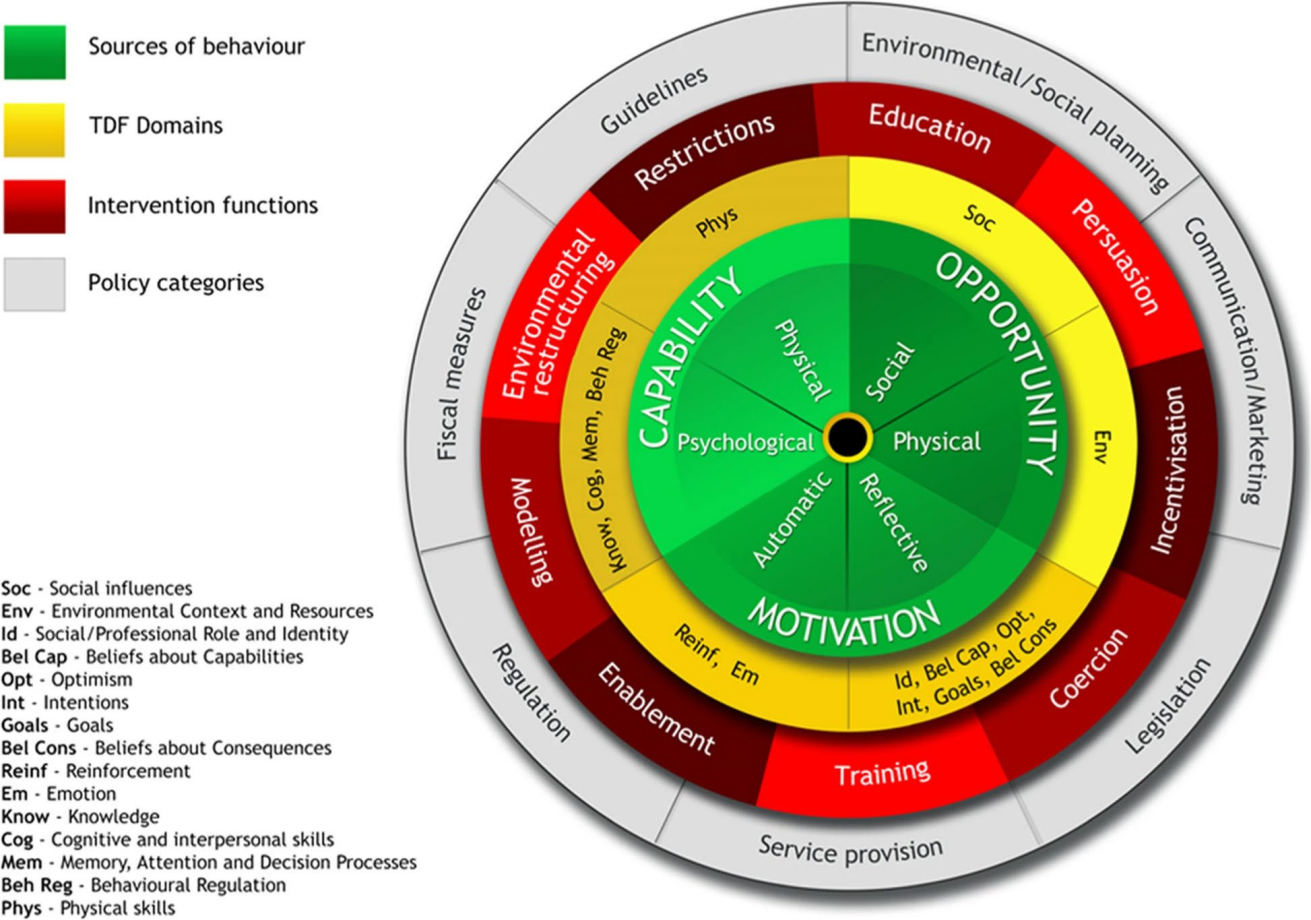
The Behaviour Change Wheel (reproduced with written permission from Michie et al. (2014)). Protected by copyright

The COM-B model is at the centre of the Behaviour Change Wheel framework, which suggests that capability, opportunity and motivation (COM) interact to influence behaviour (B) (Fig. 1). There are six COM-B components: physical capability, psychological capability, social opportunity, physical opportunity, reflective motivation and automatic motivation. There are three layers surrounding the COM-B model. The Theoretical Domains Framework, containing 14 domains, is an integrative framework developed for cross-disciplinary implementation and other behaviour change research [45]. It was added to the Behaviour Change Wheel (forming the second layer in Fig. 1) to allow deeper exploration of the factors influencing behaviour change. The following two layers represent the intervention functions (third layer, Fig. 1), and policy categories (fourth layer, Fig. 1). The Behaviour Change Wheel [43] provides an APEASE (Affordability, Practicability, Effectiveness and Cost-effectiveness, Acceptability, Side effects/safety and Equity) criteria for researchers to consider when selecting relevant intervention options and content. After information is obtained from the Behaviour Change Wheel, behaviour change techniques can be identified via the behaviour change technique taxonomy [46]. The COM-B model and Behaviour Change Wheel has been successfully used in different contexts to inform and design behaviour change intervention, including providing sexual counselling [47], decreasing sedentary behaviour in the workplace [48] and more specifically, increasing PA [49–53].

The aim of this study was to co-design the Girls Active Project (GAP) intervention to increase PA levels of adolescent females in a single-sex, females-only, designated disadvantaged post-primary school, using the Behaviour Change Wheel (BCW) and Public and Patient Involvement (PPI). To our knowledge, this is the first study that has used this methodological approach.

## Methods

### Study design

The ‘Behaviour Change Wheel Guide’ was followed for this intervention development study [43]. A mixed-method approach was used via questionnaires and semi-structured focus groups with a sample of adolescent females (aged 12 to 18) and teachers to understand the target behaviour. Discussion groups with a PPI Youth Advisory Group (adolescent females aged 15 to 17) were used to co-design the school-based PA intervention through identifying intervention options and content. Ethical approval for this study was granted by Dublin City University Research Ethics Committee (DCUREC/2019/005).

### Recruitment

#### School

One single-sex, females-only, designated disadvantaged post-primary school in Dublin, Ireland was recruited. In Ireland, a classification system known as DEIS (Delivering Equality of Opportunity in Schools) is used by the Department of Education to indicate that the school is based in a community at risk of disadvantage and social exclusion [54]. Taking into account the normal attrition rates in such studies, an email of invitation to participate in the Girls Active Project was sent to the school principal of nine single-sex, females-only, DEIS post-primary schools in Dublin, and a follow-up phone call was made to explain the purpose of the project. The Girls Active Project involved two phases; intervention development and a feasibility trial of the intervention. Three schools showed interest and two accepted the invitation. Just one school retained, thus the scope of the project was widened to include more students. The participating school had 6 year groups and students were aged from 12 to 18.

#### Research participants and PPI contributors

Research and Public and Patient Involvement (PPI) are separate activities, differing in whether the public are involved as ‘research participants’ or as ‘contributors to the research process’, i.e. PPI contributors. Using the terms ‘focus group’ and ‘discussion group’ to differentiate between research and public involvement methods has contributed to improved clarity in research [55]. For the purposes of this study, the following definitions by Doria et al. (2018) were used:

‘Focus groups’ refer to research activities. In focus groups, researchers collect data by speaking with a group of research subjects about their experiences. Researchers use this information to answer research questions and share their findings in academic journals and gatherings.

‘Discussion groups’ refer to engagement or involvement activities. Discussion groups are a way for the public to help plan research projects. Their contributions are not treated as research data, but instead they help make decisions that shape the research process.

##### Research participants

All students in the school were invited to complete a short anonymous questionnaire during school hours via an electronic portable device. Since the questionnaire was of low risk to students, parental/guardian opt-out (passive) consent was proposed [56, 57]. Conversations took place with the school regarding parental/guardian opt-out consent where they welcomed this process. A week prior to data collection, every student was provided with an information sheet and a parental/guardian opt-out consent form to read and take home to parents/guardians. Students and parents/guardians had time to understand the information and make an informed decision. At the start of the questionnaire, students were asked to provide their assent. Students were excluded from the study if they had not provided assent or if their parent/guardian had completed the parental/guardian opt-out written consent form. Six Junior Cycle students (Years 1 to 3) and six Senior Cycle students (Years 4 to 6) were selected by the physical education teacher and invited to take part in an audio-recorded focus group facilitated by the researcher (SMQ) during school hours. Each focus group aimed to include two students from each year group and a mix of perceived PA levels, based on, for example whether the students participated in physical education class or were involved in sports teams or not. It was believed that the physical education teacher was best placed to have knowledge regarding this. Each student provided written informed assent and parental/guardian consent prior to taking part in the focus group. A Girls Active Project ‘Steering Committee’ was established in the school. The physical education teacher provided information sheets to members of school staff and parents from the parents council inviting them to join the Steering Committee. All Steering Committee members provided written informed consent before taking part in data collection. More information on these data collection methods are explained below (Behaviour Change Wheel Step 4).

##### PPI contributors

Eight Transition Year students (aged 15 to 17) joined a Girls Active Project ‘Youth Advisory Group’ and participated in five PPI discussion groups to co-design the school-based PA intervention. In Ireland, Transition Year is a one-year school programme that can be taken in the year after the Junior Cycle and before the two-year Leaving Certificate programme [58]. It is not a standard academic year. The year is designed around giving students life skills and a more hands-on aspect to learning. Written informed assent and parental/guardian consent was obtained from the eight Youth Advisory Group members (PPI contributors). The researcher (SMQ) facilitated five discussion groups during school hours, each lasting 80 min (a double-class). A guide on how to actively involve young people in research [59] was adhered to. The school principal and staff designated time and space for the discussion groups and ensured that the students were available.

### The behaviour change wheel

The step-by-step method for designing behaviour change interventions in the Behaviour Change Wheel guide was used [43]. This included three stages (eight steps) in the intervention design process: understand the behaviour (Stage 1), identify intervention options (Stage 2) and identify content and implementation options (Stage 3).

#### Stage 1: understand the behaviour (steps 1 to 4)

The need to increase moderate to vigorous PA levels in adolescent females is already well established in the literature [1, 24]. The first PPI discussion group was set-up with the Youth Advisory Group to introduce the researcher (SMQ), explain the specified target behaviour (increase PA levels), the purpose of the Girls Active Project and their role as contributors to the research process. Step 4 of the Behaviour Change Wheel involved identifying what needed to change. The Behaviour Change Wheel guide [43] recommends that data is collected from multiple sources and from more than one method to understand what needs to change, as this will increase confidence in the analysis. Drawing from both the COM-B model and Theoretical Domains Framework, this study used a mixed-method approach (questionnaires and semi-structured focus groups) with multiple research participants (students and teachers).

##### Questionnaires

The student questionnaire took approximately 10 min to complete. It was anonymous and captured information including their age, year group (year 1 to 6) and current PA levels via the PACE+ scale [60]. The PACE+ scale is a two-item PA questionnaire for assessing attainment of PA guidelines and has been previously validated in a sample of 10–18 year old Irish youth [61]. This instrument has been used with adolescents both nationally [8] and internationally [62] and has acceptable reliability. Michie and colleagues developed a COM-B Self-Evaluation Questionnaire (COM-B-Qv1) for individuals relevant to the COM-B model [43]. It contains 23 different statements categorised into ‘capability’, ‘opportunity’ and ‘motivation’. The individual responds with a yes or no answer. For example, ‘For me to do physical activity at school, I would have to … have more physical strength, e.g. build up muscles for demanding physical work’. Its aim is to gain an insight into what it would take for the individual to change their behaviour. Similar to Murtagh et al. (2018) where they used the Behaviour Change Wheel to design the components of a community-based intervention to improve adolescent female’s PA, the research team ensured that all of the examples provided in the COM-B-Qv1 were relevant to the target behaviour (PA) and target audience (adolescents aged 12–18). To gain a further understanding into the factors influencing students’ participation in PA, the research team replaced the yes or no answer with a five-point Likert scale from 1 (strongly disagree) to 5 (strongly agree), similar to that used by Ellis et al. (2019), when identifying factors influencing postnatal PA [50]. The questionnaire was piloted amongst the Youth Advisory Group at the first discussion group. They understood the questions and no amendments were necessary based on their feedback. The Youth Advisory Group helped to administer the questionnaires using electronic portable devices over a one-day period, during which the researcher (SMQ) and the Youth Advisory Group visited each classroom individually. Teachers and/or Special Needs Assistants were present if any child required assistance due to reading or physical difficulties. In addition to the COM-B-Qv1, Michie et al. (2014) developed a COM-B Behavioural Diagnosis Form (COM-B-D) for groups/populations. The research team made the same slight adaptations to the COM-B-D and administered it to the Steering Committee via a paper-based questionnaire. These questionnaires along with all other data collection tools used can be found in the attached Supplementary Information File 1.

##### Focus groups

Three semi-structured, audio-recorded focus groups each lasting between 30 to 50 min were facilitated by the researcher (SMQ) during school hours; one with six Junior Cycle students, one with six Senior Cycle students and one with the Steering Committee. The aim of the focus groups were to further investigate which of the COM-B components might influence adolescent females’ PA behaviour at school. Questions asked in the focus groups were developed using the COM-B model and the Theoretical Domains Framework. The Theoretical Domains Framework has been validated for use in behaviour change and implementation research [45]. The topic guide was assessed by the Youth Advisory Group at the first discussion group. They understood the questions and no amendments were necessary based on their feedback. Handwritten notes were taken by researcher (SMQ) during each focus group.

##### Data analysis

Questionnaire data was analysed using IBM SPSS Statistics V.25, including the descriptive data analysis of participant characteristics. Physical activity (PACE+) data were scored according to recommended guidelines [60]. The mean, standard deviation (SD) and 95% confidence interval (CI) for each COM-B statement response and for each COM-B component was calculated and categorised into: agree (≥4), neutral (≥3 < 4) and disagree (< 3). The focus groups were transcribed verbatim. Participant confidentiality and anonymity was maintained by using pseudonyms [63]. The COM-B model and Theoretical Domains Framework were used as a combined deductive framework for the analysis [64]. Each transcript was read and re-read several times and coded by the researcher (SMQ). These coded transcripts were reviewed by the research team (SMQ, AS, SJB and MRS) and any discrepancies were resolved by discussion to generate a selection of factors influencing adolescent females’ PA behaviour at school.

#### Stage 2: identify intervention options (steps 5 and 6) and Stage 3: identify content and implementation options (steps 7 and 8)

The remaining four discussion groups with the Youth Advisory Group (PPI contributors) focused on Stage 2 and Stage 3 of the Behaviour Change Wheel. Lay language was used and the Youth Advisory Group were briefly taught about research and study design in a fun environment. The researcher (SMQ) intended to build rapport with the PPI contributors. They played their own music, engaged with each discussion group and shared their thoughts and unique perspectives as both adolescent females, and students attending the school. The researcher (SMQ) aimed for each discussion group to be enjoyable, and supportive, and for the PPI contributors to feel valued and heard. The researcher (SMQ) took notes throughout each discussion group. The analysed results from the questionnaires and focus groups (Step 4) were presented to the Youth Advisory Group. Following Behaviour Change Wheel guidance [43], the researcher (SMQ) and Youth Advisory Group identified potentially relevant intervention functions by linking the COM-B components relevant to the factors influencing adolescent females’ participation in PA at school, with intervention functions. Michie et al. (2014) described an ‘intervention function’ as broad categories of means by which an intervention can change behaviour. The APEASE (Affordability, Practicability, Effectiveness and Cost-effectiveness, Acceptability, Side effects/safety and Equity) criteria was used to select the most appropriate intervention functions for the Girls Active Project intervention. The next step (Step 6) in the Behaviour Change Wheel involved linking the intervention function to policy categories that are likely to be appropriate and effective in supporting each intervention function [43]. The APEASE criteria was used to select the policy categories most appropriate for the Girls Active Project intervention. Step 7 required identifying the most fitting behaviour change techniques that could result in the increase of adolescent females’ PA levels at school. A behaviour change technique is defined as “an active component of an intervention designed to change behaviour” (Michie et al., 2014, p.145). The Behaviour Change Wheel includes an extensive taxonomy of 93 consensually agreed, distinct Behaviour Change Techniques [46]. The Behaviour Change Wheel guide offers a list of behaviour change techniques that are frequently used per intervention function. Behaviour change techniques that did not meet the APEASE criteria within the context of adolescent females’ PA behaviour at school were excluded, and the most appropriate behaviour change techniques were shortlisted and decided on through discussion. The final step (Step 8) included applying the APEASE criteria to select the most appropriate modes of delivery for the Girls Active Project intervention. Intervention dimensions including content, provider, setting, recipient, intensity and duration were also deliberated and decided upon. Intervention recruitment and retention strategies were discussed and Girls Active Project posters were designed to display in the school. The Template for Intervention Description and Replication (TIDieR) checklist [65], was employed to specify details of the intervention containing the who, what, how and where of proposed intervention delivery (Supplementary Information File 3). This was discussed with members of the Steering Committee and school principal for feedback and approval. The feasibility trial was registered online: 10.17605/OSF.IO/75HWJ (date of registration: 9th December 2020).

## Results

The results of this intervention development study are presented below in accordance with the three stages (eight steps) of the Behaviour Change Wheel intervention design process: understand the behaviour (Stage 1), identify intervention options (Stage 2) and identify content and implementation options (Stage 3). Lastly, an outline of the proposed Girls Active Project intervention is provided.

### Stage 1: understand the behaviour (steps 1 to 4)

#### Research participants’ characteristics

The student questionnaire was completed by two hundred and eighty-seven students (*n* = 287). A total of 330 females were present in the school on the day of data collection, i.e., 87% of the eligible population completed the questionnaire. There were no missing data. The students ranged from first year to sixth year, aged 12 to 18 (Mean age = 14.82, SD = 1.71). A total of 1.4% reported to meet the recommended PA guideline, i.e. an average of 60 min a day of moderate to vigorous physical activity (MVPA). While almost all females achieved at least 60 min of MVPA 1 day a week (89%), just 15% achieved it five times a week. The mean number of days per week that students undertook at least 60 min of MVPA declined as they got older (age 12 = mean number of days 3.3, to age 18 = mean number of days 2.6.). Six Junior Cycle students (age range = 12 to 15) and six Senior Cycle students (age range = 15 to 18) participated in the focus groups. Seven teachers (3 male, 4 female) joined the Steering Committee and completed the questionnaire and focus group (*n* = 7).

#### What needs to change? (step 4)

Table 1 presents student responses to the COM-B statements (*n* = 287). The majority of factors were identified as being ‘neutral’, i.e., mean scores of ≥3 and < 4. ***‘Have more time’*** (physical opportunity) to be active in school was identified by the students as the strongest predictor (Mean = 4.01, 95% CI 3.91 to 4.12). This was followed by ***‘develop a habit of doing it’*** (automatic motivation) (Mean = 3.97, 95% CI 3.87 to 4.07), and ***‘feel that I want to do it enough’*** (automatic motivation) (Mean = 3.82, 95% CI 3.71 to 3.93). Students would not be more active if they *‘have more money’* (physical opportunity) (Mean = 2.99, 95% CI 2.85 to 3.12) (i.e. ‘disagree’ mean score < 3). Across the six COM-B components, ‘automatic motivation’ was the strongest predictor to them being active at school with an overall mean score of 3.78 (95% CI 3.69 to 3.87), followed by ‘reflective motivation’ (Mean = 3.76, 95% CI 3.67 to 3.85).

**Table 1:** COM-B Questionnaire statement responses by students (*n* = 287)

Questionnaire statement*			Questionnaire Response (%)					
**For me to do physical activity at school, I would have to …**	**Mean (SD)**	**95% CI**	**1**	**2**	**3**	**4**	**5**	Categorisation **
Capability								
1. Know more about why it is important, e.g. have a better understanding of the benefits of exercising more	3.17 (0.99)	3.06 to 3.29	4.9	20.2	35.2	32.4	7.3	Neutral
2. Know more about how to do it, e.g. have a better understanding of effective ways of exercising or being physically active	3.49 (0.99)	3.37 to 3.60	2.8	15.0	26.1	42.9	13.2	Neutral
3. Have better physical skills, e.g. learn different exercises or movements to help me be physically active	3.49 (1.02)	3.37 to 3.61	3.5	14.3	26.1	41.8	14.3	Neutral
4. Have better mental skills, e.g. learn how to reason more effectively	3.25 (1.04)	3.13 to 3.38	4.5	20.9	29.3	35.2	10.1	Neutral
5. Have more physical strength, e.g. build up muscles for demanding physical work	3.19 (1.13)	3.06 to 3.32	7.0	23.0	26.5	31.4	12.2	Neutral
6. Have more mental strength, e.g. develop stronger resilience against barriers to being more active	3.37 (1.03)	3.25 to 3.49	2.4	22.0	24.4	39.0	12.2	Neutral
7. Overcome physical limitations, e.g. to get around problems of stature of disability	3.17 (1.06)	3.05 to 3.29	7.3	19.5	29.6	35.9	7.7	Neutral
8. Overcome mental obstacles, e.g. develop stronger resilience against the temptation to not exercise	3.49 (1.06)	3.37 to 3.61	3.8	16.0	23.0	41.5	15.7	Neutral
9. Have more physical stamina, e.g. develop a great capacity to maintain physical effort	3.56 (0.97)	3.45 to 3.67	3.5	10.8	25.1	47.4	13.2	Neutral
10. Have more mental stamina, e.g. develop a greater capacity to maintain mental effort	3.49 (0.97)	3.38 to 3.61	2.8	13.6	27.2	44.3	12.2	Neutral
*Mean score: psychological capability component* (statements: 1,2,4,6,8,10)	3.38 (0.70)	3.30 to 3.46						Neutral
*Mean score: physical capability component* (statements: 3,5,7,9)	3.35 (0.73)	3.27 to 3.44						Neutral
Opportunity								
11. Have more time to do it, e.g. create dedicated time during the day	4.01 (0.91)	3.91 to 4.12	1.0	6.3	15.3	44.9	32.4	Agree
12. Have more money, e.g. be given or earn funds to support the behaviour	2.99 (1.17)	2.85 to 3.12	7.7	33.1	24.4	22.6	12.2	Disagree
13. Have the necessary materials, e.g. acquire better clothes/shoes/other equipment for the task	3.59 (1.07)	3.47 to 3.72	4.9	10.8	24.0	40.8	19.5	Neutral
14. Have it more easily accessible, e.g. easier access to facilities	3.60 (0.98)	3.49 to 3.71	1.7	12.9	26.5	41.5	17.4	Neutral
15. Have more people around me doing it, e.g. be part of a “crowd” who are doing it	3.26 (1.20)	3.12 to 3.40	9.8	19.2	20.2	36.9	13.9	Neutral
16. Have more triggers to prompt me, e.g. have more reminders at strategic times	3.30 (0.93)	3.20 to 3.41	3.5	15.0	36.2	38.3	7.0	Neutral
17. Have more support from others, e.g. have my friends or classmates behind me	3.42 (1.08)	3.30 to 3.55	4.9	15.3	28.2	35.9	15.7	Neutral
*Mean score: physical opportunity component* (statements: 11,12,13,14)	3.55 (0.73)	3.46 to 3.63						Neutral
*Mean score: social opportunity component* (statements: 15,16,17)	3.33 (0.80)	3.24 to 3.42						Neutral
Motivation								
18. Feel that I want to do it enough, e.g. feel more of a sense of pleasure or satisfaction from exercise	3.82 (0.94)	3.71 to 3.93	1.7	7.7	20.6	46.7	23.3	Neutral
19. Feel that I need to do it enough, e.g. care more about the negative consequences of not doing it	3.55 (1.03)	3.43 to 3.67	4.2	10.8	27.9	39.7	17.4	Neutral
20. Believe that it would be a good thing to do, e.g. have a stronger sense that I should do it	3.75 (0.91)	3.64 to 3.86	2.1	7.0	23.7	48.4	18.8	Neutral
21. Develop better plans for doing it, e.g. have a clearer and better developed plan for exercising regularly	3.76 (0.88)	3.66 to 3.86	1.7	5.9	24.7	49.5	18.1	Neutral
22. Develop a habit of doing it, e.g. get into a pattern of exercising regularly without having to think	3.97 (0.88)	3.87 to 4.07	1.0	5.2	18.5	46.3	28.9	Neutral
*Mean score: automatic motivation component* (statements: 18,19,22)	3.78 (0.76)	3.69 to 3.87						Neutral
*Mean score: reflective motivation component* (statements: 20,21)	3.76 (0.78)	3.67 to 3.85						Neutral

* participants responded on a scale of 1 (strongly disagree) to 5 (strongly agree)

** mean response to statement categorised as agree ≥4, neutral ≥3 < 4, disagree < 3

According to the Steering Committee (n = 7), the strongest predictors to increase adolescent females’ PA participation at school were if the students ***‘develop a habit of doing it’*** (automatic motivation) (Mean = 4.71, 95% CI 4.26 to 5.17), ***‘have more support from others****’* (social opportunity) (Mean = 4.71, 95% CI 4.26 to 5.17) and *‘****overcome mental obstacles’*** (psychological capability) (Mean = 4.71, 95% CI 4.26 to 5.17). To *‘have more money’* (physical opportunity) (Mean = 2.29, 95% CI 1.01 to 3.56) or to ‘*have the necessary materials’* (physical opportunity) (Mean = 2.57, 95% CI 2.08 to 3.07) were not regarded by the Steering Committee as predictors to students’ PA participation.

The focus group responses identified six domains in the Theoretical Domains Framework as factors influencing adolescent females’ PA behaviour at school. Using the COM-B model, psychological capability, physical opportunity, social opportunity, reflective motivation and automatic motivation were recognised as potentially important COM-B components to target. A summary of the identified domains and their associated COM-B component is provided in Table 2.

**Table 2:** Summary of behavioural diagnosis using the Theoretical Domain Framework and COM-B components over the three focus groups (*n* = 19)

TDF Domain	Domain Definition	Summary	Participant Quote	COM-B Component
Behavioural regulation	Anything aimed at managing or changing objectively observed or measured actions	Adolescent females needed to have systems in place that they could use for monitoring whether they participated in PA. They needed to action-plan, self-monitor and adopt procedures or ways of working that encouraged them to do PA.	“[we have] no [reminders], unless, you’re part of a team and you’ve training” (Student B, Senior Cycle). “I think reminders would work. Like get the teachers to write ‘PE tomorrow’ on the board. Or get the prefects to remind the class to remind them to bring in the gear for the next day” (Student F, Junior Cycle).	Psychological Capability
Environmental context and resources	Any circumstance of a person’s situation or environment that discourages or encourages the development of skills and abilities, independence, social competence, and adaptive behaviour.	Some of the students felt that time, the changing of uniforms for PE class, not having PE as an option and the lack of sport options available in the school, were barriers to being active. The Steering Committee felt the school offered many opportunities for the students to be active.	“it’s a massive effort to change into PE tracksuits, so [students] just say they forgot them and not do PE” (Student D, Junior Cycle). “there isn’t as many [sport] teams for seniors” (Student C, Senior Cycle). “if you do honours maths you can’t do PE, cause its during that time” (Student B, Senior Cycle). “there’s very little we don’t have” [and] “there is no day after school that the hall is free, and the lunch times too” (Teacher B, Steering Committee). “we have lots of options” (Teacher F, Steering Committee).	Physical Opportunity
Social influence	Those interpersonal processes that can cause individuals to change their thoughts, feelings, or behaviours.	Adolescents needed social support from their friends, family and classmates to exercise, and the opportunity to be part of a group and provide encouragement. Many of the females felt nervous about exercising alone and worried about how they would be perceived by their peers.	“I wanted to join the fitness club, but none of my friends would do it” (Student A, Junior Cycle). “you won’t do it if there’s someone in the group you don’t get on with, or if none of your friends are doing it” [and] “I don’t want to look stupid, or have people laugh at me” (Student D, Junior Cycle) “if your friends are there with you, you have more confidence” (Student B, Junior Cycle). “I’d probably end up quitting if it weren’t for the people on the team” (Student B, Senior Cycle). “people would rather do other stuff, like hang out with your friends” (Student F, Senior Cycle). “they won’t play because they’re afraid of what their mates would say” (Teacher F, Steering Committee). “it’s always about how they look, and how they’re perceived. That’s the number one barrier from what I’ve seen” (Teacher C, Steering Committee). “I think being in a group of friends is a really positive aspect of them engaging in physical activity because we know with [Teacher G], we do our class and its groups of friends that come, and it’s a positive influence all the time” (Teacher A, Steering Committee).	Social Opportunity
Beliefs about capabilities	Acceptance of the truth, reality, or validity about an ability, talent, or facility that a person can put to constructive use.	Adolescent females lacked confidence in doing PA, especially alone. They found it difficult to try new sports or activities and became self-conscious when doing so.	“it’s difficult when you’re trying something new” (Student F, Junior Cycle). “you become very aware of yourself when you’re surrounded by girls who are very good at it, and you start to close into yourself” (Student A, Senior Cycle). “I think their confidence levels are on the floor” (Teacher C, Steering Committee). “in terms of capabilities, I think there is loads of capabilities, but they don’t often see it” (Teacher F, Steering Committee).	Reflective Motivation
Goals	Mental representations of outcomes or end states that an individual wants to achieve.	Other priorities, for example homework, exams, family and social life competed for adolescent female’s time. They struggled to balance it all and needed to feel that PA was a priority.	“when teachers give us homework, they give us a lot and like you don’t have the time to do it at home, and you want to do exercise, but you can’t because you’ve stuff at home to do” (Student B, Junior Cycle). “just trying to fit everything in, you’re supposed to get 8 h of sleep, then 3 h of study a night, and then be active as well?” (Student C, Senior Cycle). “their commitment levels just fall off, and their priorities change” (Teacher C, Steering Committee).	Reflective Motivation
Reinforcement	Increasing the probability of a response by arranging a dependent relationship, or contingency, between the response and a given stimulus.	Adolescent females needed to feel that they wanted to do PA and for it to be fun/enjoyable. They liked having choice/autonomy and being with friends. There were punishments in place for not partaking in PE at school, but felt that they wanted more positive reinforcement for them to participate in PA.	“stuff with friends. Or even if they did ‘trial classes’, which would introduce new people to a sport” (Student F, Junior Cycle). “they [teachers] don’t change the sport, even if we say we don’t like it” (Student D, Senior Cycle). “it’s important to balance fun and competition – it wouldn’t be fun without the competition either, a mix of the two is good*”* (Student A, Senior Cycle). “I think in first year, they say you have to join at least one team but that’s it. They tell us to join one team, but we’re not followed up on it” (Student E, Senior Cycle). “you’d get a bad note into your journal if you didn’t bring in your PE gear or if you refuse to take part” (Student C, Senior Cycle).	Automatic Motivation

*PA* Physical Activity, *PE* Physical Education, *TY* Transition Year

*Psychological capability: Behavioural regulation*. Many students didn’t plan when they’ll participate in PA or have a system in place that objectively managed, monitored, encouraged or regulated their PA behaviour. *“[We have] no [reminders], unless, you’re part of a team and you’ve training”* (Student B, Senior Cycle). *Physical opportunity: Environmental context and resources*. This was the only domain where there was a clear difference in opinion. Barriers were identified by students in the school context that discouraged them to participate in PA, such as a lack of options *“there isn’t as many* [sport] *teams for seniors”* (Student C, Senior Cycle) and the changing of uniforms for physical education class. The Steering Committee believed that the students had many choices and opportunities to be active at school, *“We have lots of options”* (Teacher F, Steering Committee), *“There’s very little we don’t have”* (Teacher B, Steering Committee).

*Social opportunity: Social influence*. The influence of peers and classmates was identified as both a facilitator to PA participation *“if your friends are there with you, you have more confidence”* (Student B, Junior Cycle) “*I’d probably end up quitting if it weren’t for the people on the team”* (Student B, Senior Cycle), and a barrier to PA participation *“you won’t do it if there’s someone in the group you don’t get on with, or if none of your friends are doing it”* [and] *“I don’t want to look stupid, or have people laugh at me*” (Student D, Junior Cycle). Steering Committee members also regarded peers’ influence as both a barrier, *“they won’t play because they’re afraid of what their mates would say*” (Teacher F, Steering Committee). *“It’s always about how they look, and how they’re perceived. That’s the number one barrier from what I’ve seen”* (Teacher C, Steering Committee) and a facilitator to their participation in PA, *“I think being in a group of friends is a really positive aspect of them engaging in physical activity because we know with* [Teacher G], *we do our class and its groups of friends that come, and it’s a positive influence all the time”* (Teacher A, Steering Committee).

*Reflective motivation: Beliefs about capabilities*. *“In terms of capabilities, I think there is loads of capabilities, but they don’t often see it”* (Teacher F, Steering Committee). Students’ lack of confidence deterred them from being active or to try new sports and activities. They felt self-conscious and often compared their own capabilities to that of their classmates, *“you become very aware of yourself when you’re surrounded by girls who are very good at it, and you start to close into yourself”* (Student A, Senior Cycle). *Reflective motivation: Goals.* Other commitments, such as homework and exams resulted in students not prioritising PA or setting goals to participate in PA, *“just trying to fit everything in, you’re supposed to get 8 h of sleep, then 3 h of study a night, and then be active as well?”* (Student C, Senior Cycle). The Steering Committee also acknowledged this, particularly as students got older, *“their commitment levels just fall off, and their priorities change”* (Teacher C, Steering Committee).

*Automatic motivation: Reinforcement*. Students identified negative reinforcements, such as getting a note in their school journal if they didn’t bring in their correct uniform for physical education class, but needed more positive reinforcements to participate in PA at school. They wanted to feel encouraged to participate and liked having choices. Enjoyment was critical, and a good balance between competition and fun, *“It’s important to balance fun and competition, it wouldn’t be fun without the competition either, a mix of the two is good”* (Student A, Senior Cycle). Steering Committee members believed that more positive reinforcement could be beneficial, and suggested rewards for students’ PA participation, such as ‘certificates for attendance’.

### Stage 2: identify intervention options (steps 5 and 6)

The five COM-B components (psychological capability, physical opportunity, social opportunity, reflective motivation and automatic motivation) that were identified as potentially important (Step 4) were mapped directly onto nine intervention functions. After application of the APEASE criteria, seven intervention functions (education, persuasion, incentivisation, training, environmental restructuring, modelling and enablement) and four policy categories (communication/marketing, guidelines, environmental/social planning and service provision) were selected. Intervention functions (coercion and restriction) and policy categories (fiscal measures, regulation and legislation) were not considered acceptable or practical in this study context. More information on this selection process is provided in the Supplementary Information File 2.

### Stage 3: identify content and implementation options (steps 7 and 8)

The majority of the 93 Behaviour Change Techniques listed were identified as potentially relevant. After careful deliberation and application of the Behaviour Change Wheel APEASE criteria, 21 were selected as most appropriate to increase adolescent females’ PA levels in this school setting. The 21 Behaviour Change Techniques, their definitions and strategies on how they will be operationalised are presented in Table 3. The preferred mode of delivery was ‘face-to-face’ at a group-level via peer-delivered exercise classes. This was supported by ‘distance’ delivery at a population-level. Details of the Girls Active Project intervention and benefits of participating in PA will be communicated to the students, parents/guardians and school staff via the school’s digital media (social media), newsletter and posters displayed in the school. This aims to provide information about the Girls Active Project, and also encourage social support from students’ families, peers and school staff. For mode of delivery (Step 8), a summary of the APEASE criteria selection process and intervention dimensions can be found in the Supplementary Information File 2.

**Table 3:** Selected behaviour change techniques for the Girls Active Project intervention and intervention strategy

Selected Behaviour Change Technique (code) Definition^a^	Girls Active Project Intervention strategy
**Goal setting (behaviour) (1.1)** Set or agree a goal defined in terms of the behaviour to be achieved.	Intervention recipients set a goal to attend a weekly after-school PA programme where they partake in an exercise class.
**Action planning (1.4)** Prompt detailed planning of performance of the behaviour (must include at least one of context, frequency, duration and intensity). Context may be environmental (physical or social) or internal (physical, emotional or cognitive).	Recipients are asked to plan to attend the weekly after-school PA programme.
**Monitoring of behaviour by others without feedback (2.1)** Observe or record behaviour with the person’s knowledge as part of a behaviour change strategy.	Recipients’ behaviour is monitored at the weekly after-school PA programme.
**Social support (practical) (3.2)** Advise on, arrange, or provide practical help (e.g., from friends, relatives, colleagues, ‘buddies’ or staff) for performance of the behaviour.	Practical help is provided to recipients at the after-school PA programme. Parents/guardians are advised (via written form) to provide practical help to facilitate their daughters participation. School staff are advised (verbally) to provide practical help to facilitate students’ participation, such as volunteering to supervise the weekly after-school programme.
**Social support (emotional) (3.3)** Advise on, arrange, or provide emotional social support (e.g., from friends, relatives, colleagues, ‘buddies’ or staff) for performance of the behaviour.	Emotional social support is provided to recipients at the after-school programme. School staff and parents/guardians are advised to provide encouragement and emotional support to facilitate PA participation.
**Instruction on how to perform a behaviour (4.1)** Advise or agree on how to perform the behaviour.	Project Leaders (intervention providers) advise on how to perform PA at the after-school programme.
**Information about health consequences (5.1)** Provide information (e.g., written, verbal, visual) about health consequences of performing the behaviour.	Information on the benefits of regular participation in PA are explained to intervention recipients (verbally).
**Monitoring of emotional consequences (5.4)** Prompt assessment of feelings after attempts at performing the behaviour.	Each week, intervention recipients are asked how they feel after the exercise class.
**Demonstration of the behaviour (6.1)** Provide an observable sample of the performance of the behaviour, directly in person or indirectly e.g., via film, pictures, for the person to aspire to or imitate.	Project Leaders (intervention providers) demonstrate how to perform PA at the after-school programme.
**Prompts/cues (7.1)** Introduce or define environmental or social stimulus with the purpose of prompting or cueing the behaviour. The prompt or cue would normally occur at the time or place of performance.	Intervention recipients receive emails from the school to remind them to participate in the weekly after-school PA programme. School staff and parents/guardians are advised to provide regular verbal reminders. These prompts reinforce other BCTs by reminding recipients of the benefits of PA (5.1) and action planning (1.4).
**Behavioural practice/rehearsal (8.1)** Prompt practice or rehearsal of the performance of the behaviour one or more times in a context or at a time when the performance may not be necessary, in order to increase habit and skill.	Recipients practice PA at each exercise class in the after-school programme.
**Habit formation (8.3)** Prompt rehearsal and repetition of the behaviour in the same context repeatedly so that the context elicits the behaviour.	Recipients repeatedly participate in PA on a weekly basis at the after-school programme.
**Generalisation of a target behaviour (8.6)** Advise to perform the wanted behaviour, which is already performed in a particular situation, in another situation.	Intervention recipients are advised to be active during the week too. This generalisation of PA also reinforces the BCT (information about health consequences, code 5.1) by reminding recipients of the benefits of PA.
**Credible source (9.1)** Present verbal or visual communication from a credible source in favour of or against the behaviour.	Present verbal communication by the research team explaining key benefits of regular PA for health.
**Material incentive (behaviour) (10.1)** Inform that money, vouchers or other valued objects will be delivered if and only if there has been effort and/or progress in performing the behaviour.	Inform intervention recipients that they are entered into a raffle to win prizes (e.g. vouchers or other valued objects) if and only if there has been progress and/or an effort to participate in the after-school PA programme.
**Material reward (behaviour) (10.2)** Arrange for the delivery of money, vouchers or other valued objects if and only if there has been effort and/or progress in performing the behaviour.	Recipients that have made progress and/or an effort to participate in the after-school PA programme are entered into a raffle to win prizes (e.g. vouchers or other valued objects). Prizes are delivered to raffle-winners.
**Non-specific reward (10.3)** Arrange delivery of a reward if and only if there has been effort and/or progress in performing the behaviour.	Recipients receive a researcher signed ‘Girls Active Project Certificate of Award’ for their participation. Project Leaders (intervention providers) also receive a researcher signed ‘Girls Active Project Certificate of Achievement’ for their participation in the Girls Active Project.
**Social reward (10.4)** Arrange verbal or non-verbal reward if and only if there has been effort and/or progress in performing the behaviour.	Congratulate intervention recipients after each exercise class that they participate in.
**Non-specific incentive (10.6)** Inform that a reward will be delivered if and only if there has been effort and/or progress in performing the behaviour.	Inform intervention recipients that they will receive a (researcher) signed ‘Girls Active Project Certificate of Award’ for their participation in the Girls Active Project.
**Restructuring the social environment (12.2)** Change, or advise to change the social environment in order to facilitate performance of the wanted behaviour or create barriers to the unwanted behaviour (other than prompts/cues, rewards and punishments).	Implement the Girls Active Project peer-led, after-school programme into the school environment to facilitate PA. Include the Girls Active Project in the School PA policy to serve as the school’s commitment to support and encourage their students to participate in PA. The policy is agreed to by the principal and Steering Committee and signed by the school principal. Advise the school that it is pre-arranged for an adult (e.g., Steering Committee member, physical education teacher, school staff, parent/guardian) to supervise the weekly programme. For the feasibility study, the researcher and physical education teacher will supervise the PA programme.
**Verbal persuasion about capability (15.1)** Tell the person that they can successfully perform the wanted behaviour, arguing against self-doubts and asserting that they can and will succeed.	Empower, encourage and motivate intervention recipients to be physically active. Tell recipients that they can successfully increase their participation in PA, despite current fitness levels and/or capabilities.

^a^Based on definitions reported in Michie et al. (2014)

*Abbreviations*: *PA* Physical Activity, *PE* Physical Education, *GAP* Girls Active Project, *BCT* Behaviour Change Technique

### The Girls Active Project intervention

The proposed Girls Active Project intervention was a peer-led, after-school PA programme. Exercise classes would be delivered by Transition Year students (intervention providers known as ‘Project Leaders’) to other students (intervention recipients) on a weekly basis for 45-min after school. Classes would be supervised by an adult. Project Leaders work as a team to choose what activities they deliver (e.g. dancing, boxing, football) and change it on a weekly-basis to offer variety to the students attending. The Girls Active Project intervention aimed to be enjoyable, inclusive and supportive. Intervention recipients would have opportunities to win prizes (e.g. vouchers or other valued objects) and receive a ‘Girls Active Project Certificate’ after their participation. The completed TIDieR checklist is shown in Supplementary Information File 3. Following discussions with the school principal and physical education teacher, it was agreed that the Girls Active Project would be implemented and integrated into the school PA policy, and the intervention would be trialled the following academic term. The feasibility trial was registered online: 10.17605/OSF.IO/75HWJ (date of registration: 9th December 2020). The findings from the feasibility trial will be reported on separately.

## Discussion

This methodological paper outlines how the Behaviour Change Wheel, a comprehensive, evidence-based, theory-driven framework was systematically used in combination with a PPI approach to develop a school-based intervention to increase adolescent females’ PA levels in a single-sex, females-only, designated disadvantaged post-primary school in Ireland. This transparent intervention development process to co-design a behaviour change intervention could facilitate future replication, and may be useful for other researchers. The use of the Behaviour Change Wheel and Theoretical Domains Framework contributes to behavioural science methodology. Furthermore, the behavioural analysis performed adds to the body of knowledge on the range of factors influencing adolescent females’ PA behaviour.

### Understanding adolescent females’ physical activity participation at school

Consistent with previous research [1, 7, 8], the PA levels of adolescent females in this sample were far below recommended guidelines for optimum health. As part of Step 4 in the Behaviour Change Wheel, the aim was to systematically identify the factors influencing adolescent females’ PA behaviour at a single-sex, females-only post-primary school using the Theoretical Domains Framework and COM-B model. Data from twomethods (questionnaires and semi-structured focus groups) with two groups of stakeholders (students and teachers) were complementary; the questionnaire quantified the factors influencing adolescent females’ PA behaviour at school and the qualitative data provided in-depth perspectives explaining these factors.

In the current study, ‘having more time’ was identified by the students as the strongest predictor to them being more active at school. Previous research indicates that time constraints are a major barrier to PA participation for adolescent females [16, 18, 20]. Lack of time was cited by post-primary females as the main reason for not taking part in more activities/sport in a national study conducted in 2010 [16], followed by feelings of incompetence, not liking sport and no suitable activities offered. Students in this current study identified barriers to PA in the school environment, such as a lack of PA options and the changing into their physical education uniforms. These barriers have also been cited in previous studies [17–21]. The adolescent females that were not part of a sports-team identified a lack of self- behavioural regulation, and many of the students acquired limiting beliefs about their capabilities to do PA. Indeed, low levels of perceived competence [16, 20, 21], low levels of self-efficacy for being physically active [15, 18] or a lack of confidence in skills [17], have often been cited by adolescent females as common barriers to PA participation. Of note, research indicates that behaviour regulation and perceived competence are correlated with PA in adolescent females [66, 67]. Consistent with previous research [16, 18, 20] both the Steering Committee and students in this study recognised that social influences, particularly their peers’ influence, can be either a barrier or facilitator to PA participation. Lastly, adolescent females in this study identified a lack of positive reinforcements at school that would encourage them to participate in PA. They wanted increased choices in PA options and for PA to be 'fun' and enjoyable. Providing adolescent females with ‘youth agency’ and autonomy in PA activities, more emphasis on fun and involvement rather than skill and competition, increased choice, and offering a variety of activities to promote PA are common recommendations across the literature [18–20].

### Intervention development

Similar to two previous studies that used the Behaviour Change Wheel to design PA interventions, the process was considered time-consuming [51, 52] due to the large volume of choice. Despite this, the research team considered the Behaviour Change Wheel to be a useful framework to co-design the intervention. Given the scope of options provided, other behaviour change interventions using the Behaviour Change Wheel in the school-setting may produce different results. The discussion groups allowed the researcher (SMQ) and Youth Advisory Group (PPI contributors) to consider the full range of options and to choose the most appropriate through a systematic evaluation of theory and evidence, whilst ensuring that the range of opportunities being created were matched to the preferences of the students. The experience of capturing the views of adolescent females in the intervention design process and identifying specific strategies that respond to their interests and needs was perceived to be invaluable by the research team.

The proposed Girls Active Project intervention was a peer-led, after-school PA programme, containing 21 Behaviour Change Techniques. There is evidence to suggest that after-school PA programmes [68], changes in the school environment [30], and peer support [29] can be promising intervention strategies to increase adolescents' PA participation. Moreover, a systematic review by Ginis et al. (2013) found that peer-delivered PA interventions can lead to significant increases in PA that are similar in magnitude to increases achieved by those delivered by professionals [69].

### Strengths and limitations

#### Strengths

This paper describes a novel approach to designing a school-based PA intervention using the Behaviour Change Wheel in combination with a PPI approach. Obtaining data via a mixed-methods approach (questionnaires and focus groups) and use of multiple sources (students and teachers) strengthened our understanding of adolescent females' PA behaviour in this context. Stakeholder buy-in played a significant role in this study. The Youth Advisory Group, students and school staff, including the Steering Committee members and school principal were fully supportive of the Girls Active Project. The Youth Advisory Group in particular took ownership of their role as PPI contributors and truly engaged in the research process. A high proportion of the eligible research participants (students attending the school) completed the questionnaire. Previous research suggests that a contributing factor to a high response rate could be the use of parental/guardian opt-out consent [56, 57].

#### Limitations

A limitation of using parental/guardian opt-out consent was that ethically, no personal identifying information could be collected. This included students’ home street address, often used to identify one’s social deprivation status Individual socio-economic status data was not collected. This is a limitation to this study. This study was conducted in one single-sex, females-only, designated disadvantaged post-primary school in Ireland. These findings may not be generalisable to the wider community. The views and contributions of the eight students who joined the Youth Advisory Group may not represent the students in the school. The quantitative data was collected in a day, thus females that were absent that day did not contribute. Lastly, the PA levels of adolescent females in this sample were self-reported (subjective), which is dependent on students' recall ability [70].

## Conclusion

The PA levels of adolescent females in this sample were far below recommended guidelines for optimum health. This methodological paper outlines how the Behaviour Change Wheel, a comprehensive, evidence-based, theory-driven framework was systematically used in combination with a PPI approach to co-design a school-based intervention to increase adolescent females’ PA levels. The intervention was tailored to overcome the barriers to adolescent females’ PA participation and take account of the preferences of adolescent females. The paper describes how a mixed-method approach using the Behaviour Change Wheel and Theoretical Domains Framework can be successfully applied through a systematic process to understand the factors influencing adolescent females’ PA behaviour in the school setting. There are implications to this research. Firstly, the transparent intervention development process described could facilitate future replication and may be useful for other researchers. Secondly, the behavioural analysis performed adds to the body of knowledge on the range factors influencing adolescent females’ PA behaviour. Thirdly, this work has informed practice in the post-primary school setting - the Girls Active Project intervention has now been implemented and its feasibility study will be reported on separately.

## Supplementary Information

**Additional file 1.**

**Additional file 2.**

**Additional file 3.**

## Data Availability

The datasets during and/or analysed during the current study are available from the corresponding author on reasonable request.

## Abbreviations

GAP: Girls Active Project
PA: Physical activity
MVPA: Moderate-to-vigorous physical activity
PPI: Public and Patient Involvement
YAG: Youth Advisory Group
SC: Steering Committee
BCW: Behaviour Change Wheel
APEASE: Affordability, Practicability, Effectiveness and Cost-effectiveness, Acceptability, Side effects/safety and Equity
COM-B: Capability, Opportunity, Motivation – Behaviour
TDF: Theoretical Domains Framework
BCTs: Behaviour change techniques
