# Supplementary material for: Co-design of a school-based physical activity intervention for adolescent females in a disadvantaged community: insights from the Girls Active Project (GAP)
Source: BMC Public Health. 2022 Mar 29;22:615. doi: 10.1186/s12889-022-12635-w (PMC8966245; doi:10.1186/s12889-022-12635-w)
Supplement: Supplementary file 1 — Additional file 1. [file 12889_2022_12635_MOESM1_ESM.pdf]

Girls Active Project (GAP)

Student Questionnaire

Age: ______________ **Class/Year: __________**

School: ___________________________________ **Date: ________________**

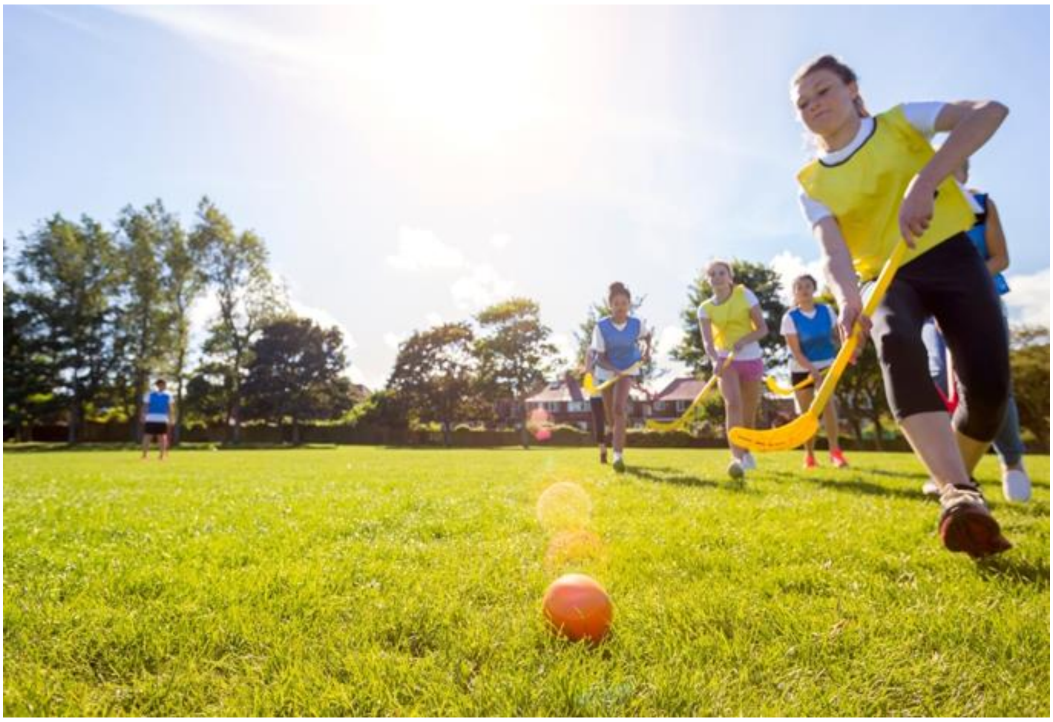

Part A: Self-Evaluation

Please read the below statements and tick the answer you feel is most appropriate to you, on a scale from 1 (strongly disagree) to 5 (strongly agree). Some of the items may look strange, but that is just because we need to include anything that might possibly apply for some people. **Please only select one answer per statement.**

Remember:

1. There are no right and wrong answers – this is not a test.
2. Please answer all the questions as honestly and accurately as you can – this is very important.

Girls Active Project

Girls Active Project (GAP)

Q1. Capability

"For me to do physical activity at school, I would have to... "

	Strongly Disagree	Disagree	Neutral	Agree	Strongly Agree
Know more about why it is important, e.g. have a better understanding of the benefits of exercising more	○	○	○	○	○
Know more about how to do it, e.g. have a better understanding of effective ways of exercising or being physically active	○	○	○	○	○
Have better physical skills, e.g. learn different exercises or movements to help me be physically active	○	○	○	○	○
Have better mental skills, e.g. learn how to reason more effectively	○	○	○	○	○
Have more physical strength, e.g. build up muscles for demanding physical work	○	○	○	○	○
Have more mental strength, e.g. develop stronger resilience against barriers to being more active	○	○	○	○	○
Overcome physical limitations, e.g. to get around problems of stature of disability	○	○	○	○	○
Overcome mental obstacles, e.g. develop stronger resilience against the temptation to not exercise	○	○	○	○	○
Have more physical stamina, e.g. develop a great capacity to maintain physical effort	○	○	○	○	○
Have more mental stamina, e.g. develop a greater capacity to maintain mental effort	○	○	○	○	○

Girls Active Project (GAP)

Q2. Opportunity

"For me to do physical activity at school, I would have to... "

	Strongly Disagree	Disagree	Neutral	Agree	Strongly Agree
Have more time to do it, e.g. create dedicated time during the day	○	○	○	○	○
Have more money, e.g. be given or earn funds to support the behaviour	○	○	○	○	○
Have the necessary materials, e.g. acquire better clothes/shoes/other equipment for the task	○	○	○	○	○
Have it more easily accessible, e.g. easier access to facilities	○	○	○	○	○
Have more people around me doing it, e.g. be part of a "crowd" who are doing it	○	○	○	○	○
Have more triggers to prompt me, e.g. have more reminders at strategic times	○	○	○	○	○
Have more support from others, e.g. have my friends or classmates behind me	○	○	○	○	○

Q3. Motivation

"For me to do physical activity at school, I would have to... "

	Strongly Disagree	Disagree	Neutral	Agree	Strongly Agree
Feel that I want to do it enough, e.g. feel more of a sense of pleasure or satisfaction from exercise	○	○	○	○	○
Feel that I need to do it enough, e.g. care more about the negative consequences of not doing it	○	○	○	○	○
Believe that it would be a good thing to do, e.g. have a stronger sense that I should do it	○	○	○	○	○
Develop better plans for doing it, e.g. have a clearer and better developed plan for exercising regularly	○	○	○	○	○
Develop a habit of doing it, e.g. get into a pattern of exercising regularly without having to think	○	○	○	○	○

Health Research Board

Girls Active Project

DCU

Girls Active Project (GAP)

Part B: Physical Activity

Read the following four statements before answering the two questions below.

Physical activity is any bodily movement. It can be done at different levels of effort:

<u>Moderate Effort</u> makes your heart rate and breathing rate faster than normal. You may also sweat a little. Brisk walking and jogging are good examples.

<u>Vigorous Effort</u> makes your heart rate much faster and you have to breathe deeper and faster than normal. You will probably sweat. Playing football or tennis are good examples.

Physical activity includes: Exercises - Weight training, aerobics, jogging, dancing, etc. Sports - Hurling, football, athletics, swimming, etc. General - Brisk walking, washing the car, walking or cycling to school, etc.

Please try to think carefully and be as accurate as possible with your answers. For these next two questions, add up all the time you spend in physical activity each day. Only include activities of either MODERATE or VIGOROUS effort (as described above).

Q5. Over the past 7 days, on how many days were you physically active for a total of at least 60 minutes per day? <u>Please tick one number.</u>

0 days	1 day	2 days	3 days	4 days	5 days	6 days	7 days
○	○	○	○	○	○	○	○

Q6. Over a typical or usual week, on how many days are you physically active for a total of at least 60 minutes per day? Only include activities of either MODERATE or VIGOROUS effort. <u>Please tick one number.</u>

0 days	1 day	2 days	3 days	4 days	5 days	6 days	7 days
○	○	○	○	○	○	○	○

Thank you

Girls Active Project (GAP)

Students Focus Group Topic Guide

School:	Group/Year:
Date:	Facilitator:
Total number of participants:	

Consent forms received. Focus groups will be audio-recorded and transcribed. There are no right or wrong answers, this will be an informal discussion.

These questions have been developed for this target group, and behaviour (i.e. physical activity), using the behaviour change wheel guidance (Michie et al., 2014). This approach incorporates all the COM-B (Capabilities, Opportunities, Motivation - Behaviour) components, and TDF (Theoretical Domain Framework) domains.

1. ***Knowledge.*** Do you know what physical activity is?
2. ***Skills.*** Can you physically do it? Do you know how to do physical activity?
3. ***Memory, attention and decision processes****.* Do you remember to do it? Is physical activity something you usually do? (Memory, attention control – the ability to retain information, selective focus)
4. ***Behavioural regulation****.* Do you have systems that you could use for monitoring whether or not you have carried out physical activity? Are there procedures or ways of working that encourages you to do it? (self-monitoring, breaking habit, action planning – anything aimed at managing or changing objectively observed or measured actions)
5. ***Environmental context and resources****.* What aspects of the school environment (physical or resource factors – i.e. material resources, school culture, weather) influence whether or not you do physical activity? To what extent do these factors facilitate or hinder you doing physical activity?
6. ***Social influence****.* How might views/opinions of others (fellow students, teachers, friends, family) influence your decision to do physical activity? To what extent do social influences facilitate or hinder physical activity? (interpersonal processes that can cause individuals to change their thoughts, feelings or PA behaviours, e.g. social norms, social pressure, group conformity, social support)
7. ***Social role and identity.*** Do you think it is part of your 'role' to do physical activity? Is doing physical activity compatible or in conflict with your identity as a person/student/teenage girl? (a

coherent set of behaviours and displayed personalities of an individual in a social or school setting, e.g. social identity, group identity, leadership)

8. ***Beliefs about capabilities.*** Are you confident in doing it? How difficult or easy is it for you to do physical activity? (acceptance of the truth about PA ability, e.g. perceived competence, self-efficacy, self-esteem)
9. ***Optimism.*** Do you think increasing PA levels is something that can be done? How confident are you that increasing physical activity levels is possible? (confidence it will happen, desired goals will be achieved – optimism).
10. ***Beliefs about consequences.*** Do you think there are any benefits/harms of doing or not doing physical activity? What do you think will happen if you do physical activity? What do you think will happen if you don't do physical activity? (acceptance of the truth about outcomes of PA - outcome expectations, beliefs).
11. ***Intentions.*** Have you made a decision to do physical activity? (conscious decisions to perform PA, stability of intentions)
12. ***Goals.*** Is there anything else that you might want to do or achieve that might interfere with you doing PA? How much do you want to do physical activity? (end states that you want to achieve, i.e. target setting, action planning)
13. ***Reinforcement.*** Is there anything that might be reinforcing you to do physical activity? Are there any incentives for you to do physical activity? (relationship between the response and a given stimulus, e.g. rewards, punishments)
14. ***Emotion.*** Does doing physical activity provoke an emotional response for you? (i.e. fear, stress, anxiety, positive affect – complex reaction pattern involving experiential, behavioural and physiological elements).

Girls Active Project (GAP)

The following questions may be asked during the focus groups for the researchers to develop a further understanding of physical activity in the school:

- What kinds of activities do you play in physical education (PE) that are fun and make you to want to participate?
- What do you like or dislike about physical education (PE) that determines if you will participate?
- What prevents you from participating in physical activity in school?, i.e. Relevant to the school context, what are the barriers to physical activity?
- And relevant to the school context, what are the facilitators to physical activity?
- What could teachers or others do to help you enjoy physical activity more?
- Do you participate in sport to win or to be with your friends?
- Is too much of a focus on winning hard for you? Do coaches, parents and teachers think winning is more important than you do?

Recruitment and retention

- If we were to design a programme to get teenage girls more active in your school, what would increase your/their interest in taking part? For e.g. social supports, timing, content?

- What strategies could be used to attract girls who have little or no previous experience in exercise or sports participation?

Health Research Board

Girls Active Project

DCU

Girls Active Project (GAP)

Steering Committee Questionnaire

School: ________________________________

Role: __________________________________ **Date: ____________________**

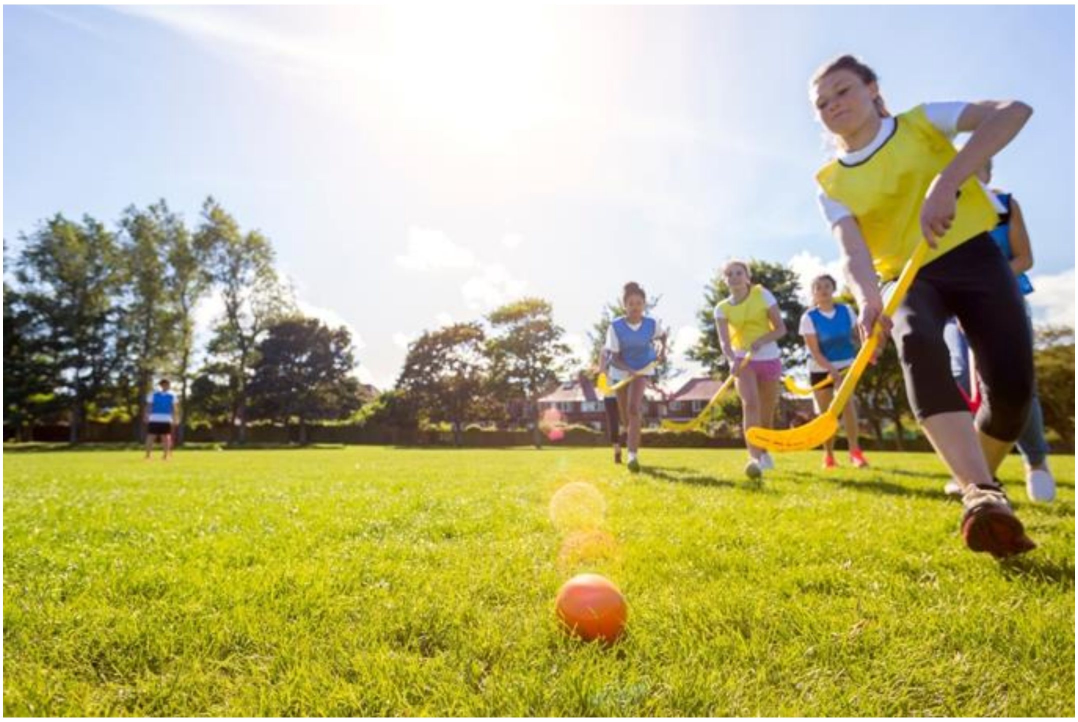

<u>Please read the below statements and tick the answer you feel is most appropriate to you, on a scale from 1 (strongly disagree) to 5 (strongly agree).</u> Some of the items may look strange, but that is just because we need to include anything that might possibly apply for some people. **Please only select one answer per statement.**

Remember:

1. There are no right and wrong answers – this is not a test.
2. Please answer all the questions as honestly and accurately as you can – this is very important.

Girls Active Project (GAP)

Q1. Capability

"When it comes to the girls doing physical activity at school, they would have to... "

	Strongly Disagree	Disagree	Neutral	Agree	Strongly Agree
Know more about why it is important, e.g. have a better understanding of the benefits of exercising more	○	○	○	○	○
Know more about how to do it, e.g. have a better understanding of effective ways of exercising or being physically active	○	○	○	○	○
Have better physical skills, e.g. learn different exercises or movements to help me be physically active	○	○	○	○	○
Have better mental skills, e.g. learn how to reason more effectively	○	○	○	○	○
Have more physical strength, e.g. build up muscles for demanding physical work	○	○	○	○	○
Have more mental strength, e.g. develop stronger resilience against barriers to being more active	○	○	○	○	○
Overcome physical limitations, e.g. to get around problems of stature of disability	○	○	○	○	○
Overcome mental obstacles, e.g. develop stronger resilience against the temptation to not exercise	○	○	○	○	○
Have more physical stamina, e.g. develop a great capacity to maintain physical effort	○	○	○	○	○
Have more mental stamina, e.g. develop a greater capacity to maintain mental effort	○	○	○	○	○

Girls Active Project (GAP)

Q2. Opportunity

"When it comes to the girls doing physical activity at school, they would have to... "

	Strongly Disagree	Disagree	Neutral	Agree	Strongly Agree
Have more time to do it, e.g. create dedicated time during the day	○	○	○	○	○
Have more money, e.g. be given or earn funds to support the behaviour	○	○	○	○	○
Have the necessary materials, e.g. acquire better clothes/shoes/other equipment for the task	○	○	○	○	○
Have it more easily accessible, e.g. easier access to facilities	○	○	○	○	○
Have more people around them doing it, e.g. be part of a "crowd" who are doing it	○	○	○	○	○
Have more triggers to prompt them, e.g. have more reminders at strategic times	○	○	○	○	○
Have more support from others, e.g. have their friends or classmates behind them	○	○	○	○	○

Q3. Motivation

"When it comes to the girls doing physical activity at school, they would have to... "

	Strongly Disagree	Disagree	Neutral	Agree	Strongly Agree
Feel that they want to do it enough, e.g. feel more of a sense of pleasure or satisfaction from exercise	○	○	○	○	○
Feel that they need to do it enough, e.g. care more about the negative consequences of not doing it	○	○	○	○	○
Believe that it would be a good thing to do, e.g. have a stronger sense that they should do it	○	○	○	○	○
Develop better plans for doing it, e.g. have a clearer and better developed plan for exercising regularly	○	○	○	○	○
Develop a habit of doing it, e.g. get into a pattern of exercising regularly without having to think	○	○	○	○	○

Thank You

Girls Active Project (GAP)

Steering Committee Focus Group Topic Guide

School:	Group/Year:
Date:	Facilitator:
Total number of participants:	

Consent forms received. Focus groups will be audio-recorded and transcribed. There are no right or wrong answers, this will be an informal discussion.

These questions have been developed for this target group, and behaviour (i.e. physical activity), using the behaviour change wheel guidance (Michie et al., 2014). This approach incorporates all the COM-B (Capabilities, Opportunities, Motivation - Behaviour) components, and TDF (Theoretical Domain Framework) domains. Similar to the questionnaire, there may be some questions that come across strange or obvious, but that is just because we need to include anything that might possibly apply for some people.

For each question, I'm asking for your personal opinion. So, in your opinion,

1. ***Knowledge.*** Do the girls know what physical activity is?
2. ***Skills.*** Can they physically do it? Do they know how to do physical activity?
3. ***Memory, attention and decision processes***. Do they remember to do it? Is physical activity something they usually do? (Memory, attention control – the ability to retain information, selective focus)
4. ***Behavioural regulation***. Do they have systems that they could use for monitoring whether or not they have carried out physical activity? Are there procedures or ways of working that encourages them to do it? (self-monitoring, breaking habit, action planning – anything aimed at managing or changing objectively observed or measured actions)
5. ***Environmental context and resources***. What aspects of the school environment (physical or resource factors – i.e. material resources, school culture, weather) influence whether or not they do physical activity? To what extent do these factors facilitate or hinder them doing physical activity?
6. ***Social influence***. How might views/opinions of others (fellow students, teachers, friends, family) influence their decision to do physical activity? To what extent do social influences facilitate or hinder physical activity? (interpersonal processes that can cause individuals to change their

thoughts, feelings or PA behaviours, e.g. social norms, social pressure, group conformity, social support)

7. ***Social role and identity.*** Do they think it is part of their 'role' to do physical activity? Is doing physical activity compatible or in conflict with their identity as a person/student/teenage girl? (a coherent set of behaviours and displayed personalities of an individual in a social or school setting, e.g. social identity, group identity, leadership)
8. ***Beliefs about capabilities***. Are they confident in doing it? How difficult or easy is it for them to do physical activity? (acceptance of the truth about PA ability, e.g. perceived competence, self-efficacy, self-esteem)
9. ***Optimism.*** Do you think they think increasing PA levels is something that can be done? How confident are they that increasing physical activity levels is possible? (confidence it will happen, desired goals will be achieved – optimism).
10. ***Beliefs about consequences.*** Do you think they think there are any benefits/harms of doing or not doing physical activity? (acceptance of the truth about outcomes of PA – outcome expectations, beliefs).
11. ***Intentions.*** Have they made a decision to do physical activity? (conscious decisions to perform PA, stability of intentions)
12. ***Goals.*** Is there anything else that they might want to do or achieve that might interfere with them doing PA? (end states that you want to achieve, i.e. target setting, action planning)
13. ***Reinforcement.*** Is there anything that might be reinforcing them to do physical activity? Are there any incentives for them to do it? (relationship between the response and a given stimulus, e.g. rewards, punishments)
14. ***Emotion.*** Do you think doing physical activity provokes an emotional response for some students? (i.e. fear, stress, anxiety, positive affect – complex reaction pattern involving experiential, behavioural and physiological elements).

Girls Active Project (GAP)

The following questions may be asked during the focus groups for the researchers to develop a further understanding of physical activity in the school:

- In your opinion, what prevents the girls from participating in physical activity in school?, i.e. Relevant to the school context, what are the barriers to physical activity?
- And relevant to the school context, what are the facilitators to physical activity?

Recruitment and retention

- If we were to design a programme to get teenage girls more active in your school, what do you think would increase their interest in taking part? For e.g. time, social supports, content?

- Do you know of any strategies that could be used to attract girls who have little or no previous experience in exercise or sports participation?

Thank You
