# Supplementary material for: Co-design of a school-based physical activity intervention for adolescent females in a disadvantaged community: insights from the Girls Active Project (GAP)
Source: BMC Public Health. 2022 Mar 29;22:615. doi: 10.1186/s12889-022-12635-w (PMC8966245; doi:10.1186/s12889-022-12635-w)
Supplement: Supplementary file 2 — Additional file 2. [file 12889_2022_12635_MOESM2_ESM.pdf]

Girls Active Project (GAP)

Discussion Groups: Youth Advisory Group
Co-Design of the Girls Active Project intervention

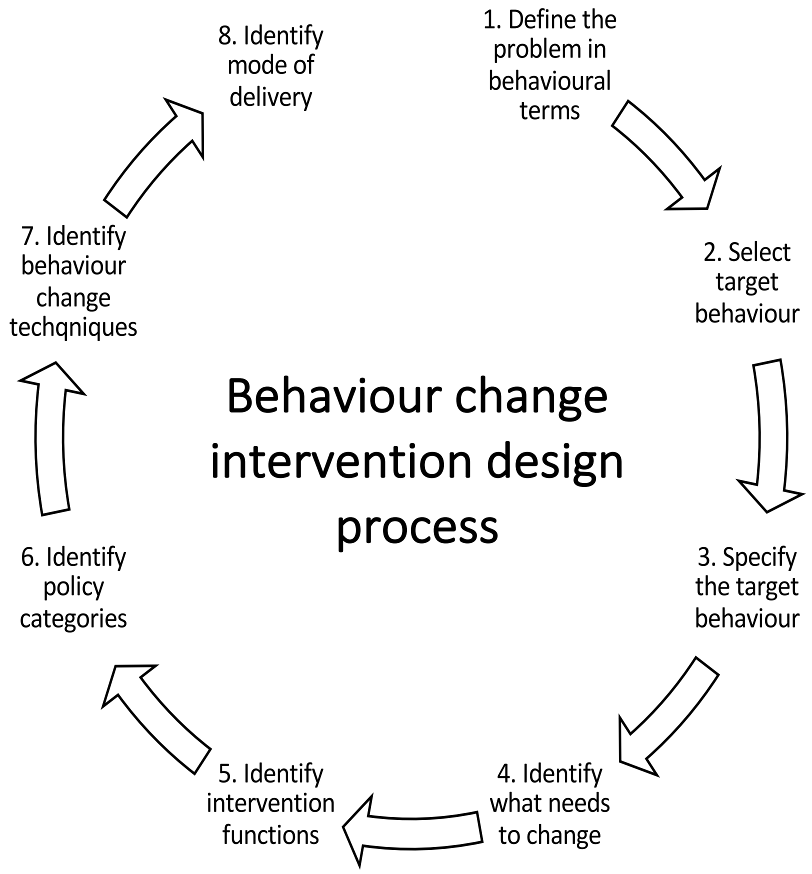

Figure: The Behaviour Change Wheel Intervention 8-Step Design Process

Reference: Michie S, Atkins L, West R. The Behaviour Change Wheel. A guide to designing interventions. London: Silverback Publishing; 2014.

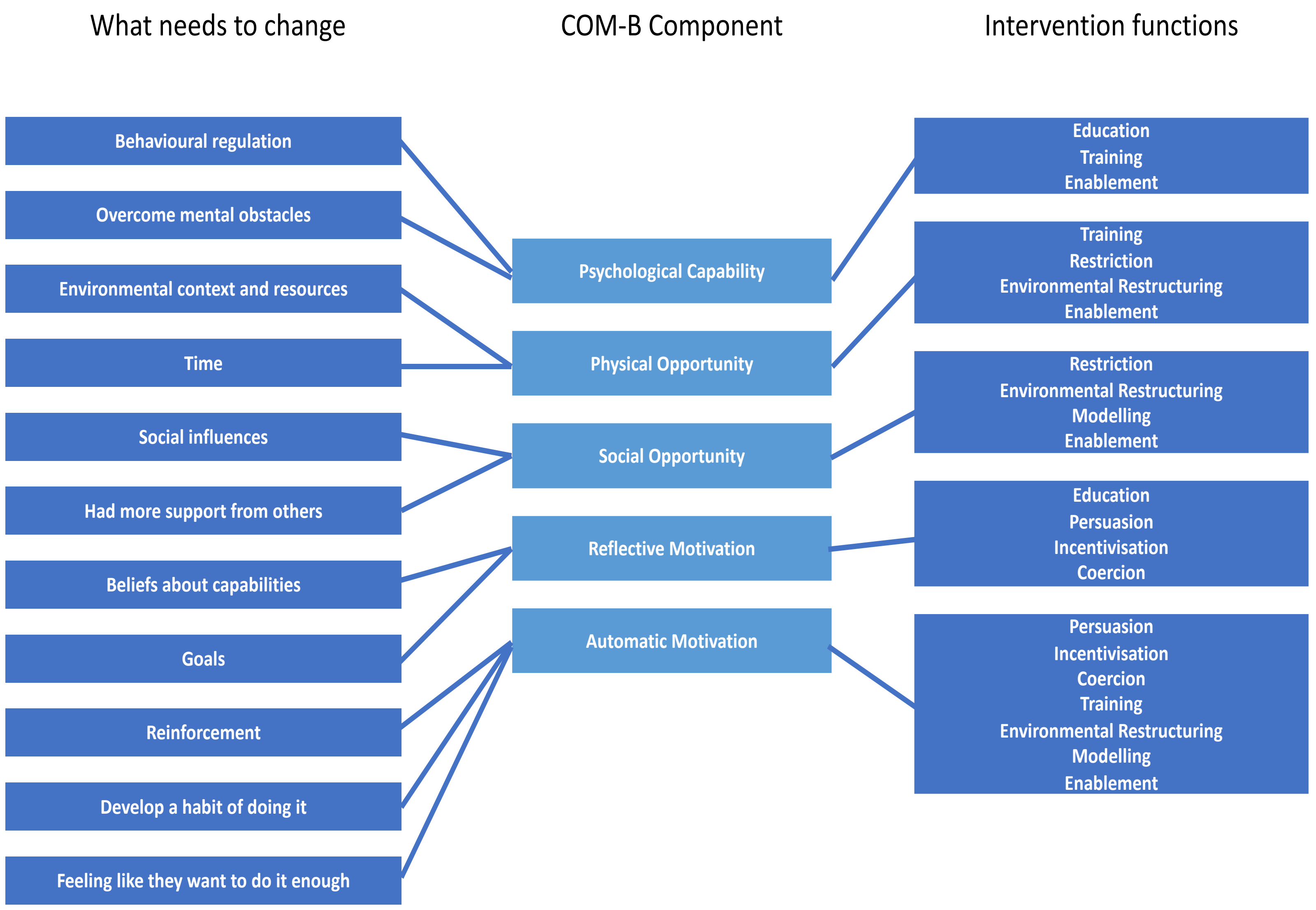

Figure: links between the Theoretical Domains Framework and COM-B model (Step 4) and intervention functions (Step 5)

Table: Applying APEASE criteria to guide intervention function selection (Step 5)

**Intervention function**<br>*Definition**	**Is the intervention function affordable, practical, effective (and cost-effective), acceptable, safe and equitable?**
**Education**<br>*Increasing knowledge or understanding*	Yes
**Persuasion**<br>*Using communication to induce positive or negative feelings or stimulate action*	Yes
**Incentivisation**<br>*Creating an expectation of reward*	Yes
**Coercion**<br>*Creating an expectation of punishment or cost*	Unlikely to be acceptable or practical
**Training**<br>*Imparting skills*	Yes
**Restriction**<br>*Using rules to reduce the opportunity to engage in the target behaviour (or to increase the target behaviour by reducing the opportunity to engage in competing behaviours)*	Not practical or acceptable
**Environmental restructuring**<br>*Changing the physical or social context*	Yes, in the school-setting
**Modelling**<br>*Providing an example for people to aspire to or imitate*	Yes
**Enablement**<br>*Increasing means/reducing barriers to increase capability (beyond education and training) or opportunity (beyond environmental restructuring)*	Yes

*Based on definitions reported in Michie et al. (2014)

Table: Matrix of links between identified intervention functions and policy categories (Step 6)

	**Girls Active Project Intervention functions**						
**Policy Categories**	Education	Persuasion	Incentivisation	Training	Environmental restructuring	Modelling	Enablement
Communication/marketing	■	■	■			■	
Guidelines	■	■	■	■	■		■
Fiscal Measures			■	■	■		■
Regulation	■	■	■	■	■		■
Legislation	■	■	■	■	■		■
Environmental/social planning					■		■
Service provision	■	■	■	■		■	■

Definitions: 'Communication/marketing' (using print, electronic, telephonic or broadcast media); 'guidelines' (creating documents that recommend or mandate practice, this includes all changes to service provision); 'fiscal' (using the tax system to reduce or increase the financial cost); 'regulation' (establishing rules or principles of behaviour or practice); 'legislation' (making or changing laws); 'environmental/social planning' (designing and/or controlling the physical or social environment); 'service provision' (delivering a service) (Michie et al., 2014, p.135)

Health Research Board

Girls Active Project

DCU

Girls Active Project (GAP)

Table: Selected modes of delivery used in the Girls Active Project intervention (Step 8)

Mode of delivery				Girls Active Project Intervention
				Is this mode of delivery affordable, practical, effective/cost-effective, acceptable, safe and equitable in the context of increasing adolescent females' physical activity levels in the school setting?
Face-to-face	Individual			Unlikely to be practical
	Group			Yes
Distance	Population-level	Broadcast media	TV	Unlikely to be affordable, practical, or equitable
			Radio	Unlikely to be affordable, practical, or equitable
		Outdoor media	Billboard	Unlikely to be affordable, practical, or equitable
			Poster	Yes
		Print media	Newspaper	Yes (school newsletter)
			Leaflet	Unlikely to be effective/cost-effective
		Digital media	Internet	Yes (school social media platforms)
			Mobile phone app	Unlikely to be affordable or equitable
	Individual-level	Phone	Phone helpline	Unlikely to be affordable, practical, acceptable, or equitable
			Mobile phone text	Unlikely to be practical, acceptable, or equitable
		Individually accessed computer programme		Unlikely to be affordable, practical, or equitable

Table: Intervention dimensions, their definitions, and their application in the Girls Active Project intervention (Step 8)

**Intervention Dimension**<br>*Definition**	**Girls Active Project Intervention**
**Mode of delivery**<br>*How it is delivered*	Face-to-face via group exercise classes at an after-school programme. Distance at a population-level via the school's digital media (social media), newsletter and posters.
**Content**<br>*What is delivered*	Exercise classes vary from week-to-week, e.g., boxing, dancing, circuits, high intensity interval training (HIIT), etc.
**Provider**<br>*Who delivers it*	Project Leaders (Transition Year students).
**Setting**<br>*Where it is delivered*	After-school programme delivered on school grounds.
**Recipient**<br>*To whom it is delivered*	Adolescent females attending the school. Recruitment via letters home, emails, and the school's social media platforms.
**Intensity**<br>*Over how many contacts it is delivered*	45 minutes on a weekly basis (Tuesdays at 4pm) for one academic school-term (excluding holidays), i.e., approximately 12 classes.
**Duration**<br>*Over what period of time it is delivered*	January to May (5 months).

*Based on definitions reported in Michie et al. (2014)
