# Supplementary material for: Co-design of a school-based physical activity intervention for adolescent females in a disadvantaged community: insights from the Girls Active Project (GAP)
Source: BMC Public Health. 2022 Mar 29;22:615. doi: 10.1186/s12889-022-12635-w (PMC8966245; doi:10.1186/s12889-022-12635-w)
Supplement: Supplementary file 3 — Additional file 3. [file 12889_2022_12635_MOESM3_ESM.docx]

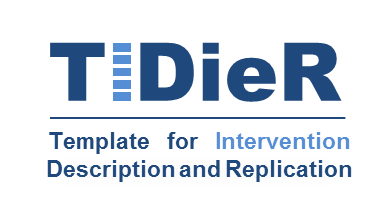


**The TIDieR (Template for Intervention Description and Replication) Checklist**

Information to include when describing an intervention and the location of the information

| **Item number** | **Item** |  |
| --- | --- | --- |
|  |  |  |
|  | **BRIEF NAME** |  |
| **1.** | Provide the name or a phrase that describes the intervention. | The Girls Active Project: a peer-led, after-school physical activity intervention |
|  | **WHY** |  |
| **2.** | Describe any rationale, theory, or goal of the elements essential to the intervention. | The Girls Active Project intervention was developed using the Behaviour Change Wheel, in combination with Public and Patient Involvement and aims to increase adolescent females’ physical activity levels. |
|  | **WHAT** |  |
| **3.** | Materials: Describe any physical or informational materials used in the intervention, including those provided to participants or used in intervention delivery or in training of intervention providers. Provide information on where the materials can be accessed (e.g. online appendix, URL). | The Girls Active Project intervention includes intervention providers (Transition Year students, adolescent females aged 15 to 17, known as ‘Project Leaders’) delivering exercise classes to other students (intervention recipients) on a weekly basis for 45-minutes after-school. The Project Leaders work as a team to choose what activities (content) they deliver (e.g. dancing, boxing, football) and change it on a weekly-basis to offer variety. |
| **4.** | Procedures: Describe each of the procedures, activities, and/or processes used in the intervention, including any enabling or support activities. | The Girls Active Project intervention consists of the delivery of 21 Behaviour Change Techniques. The school staff will support Project Leaders to deliver the school-based intervention. Details of the Girls Active Project intervention and benefits of participating in PA will be communicated to the students, parents/guardians and school staff via the school’s digital media (social media), newsletter and posters. This aims to provide information about the Girls Active Project intervention, and also encourage social support from students’ families, peers and school staff. Class attendance will be monitored. Project Leaders will be required to complete a ‘Project Leader logbook’ after each exercise class. Intervention recipients would have opportunities to win prizes (e.g. vouchers) and a Girls Active Project ‘Certificate of Award’ will be rewarded to intervention recipients after their participation in the after-school PA programme. Project Leaders (intervention providers) also receive a Girls Active Project ‘Certificate of Achievement’ for their involvement in the Girls Active Project intervention. |
|  | **WHO PROVIDED** |  |
| **5.** | For each category of intervention provider (e.g. psychologist, nursing assistant), describe their expertise, background and any specific training given. | Intervention providers are Transition Year students (adolescent females aged 15 to 17) attending the school. They would voluntarily become a Girls Active Project ‘Project Leader’. If applicable, their time as Project Leaders will contribute towards their ‘Gaisce Award’ (a national award part of a self-directed personal development programme). The Project Leaders will be briefed by the researcher and physical education teacher of their role in the intervention. Training for the intervention providers (Project Leaders) consists of workshops organised with the physical education teacher and researcher. Project Leaders are informed about the class structure and Project Leaders decide what activities they will deliver each week. Each 45-minute class will follow a similar structure: a) welcome and introductions made to intervention recipients and mention the purpose of the Girls Active Project, b) intervention recipients given a chance to contribute and ask questions; c) exercises explained and demonstrated, and intervention recipients given a chance to practise the exercises; d) intervention recipients congratulated for participating and reminded about next week’s class. All Project Leaders will work as team to deliver the intervention. The researcher and physical education teacher will be present at each exercise class to supervise and provide additional support if required. |
|  | **HOW** |  |
| **6.** | Describe the modes of delivery (e.g. face-to-face or by some other mechanism, such as internet or telephone) of the intervention and whether it was provided individually or in a group. | The Girls Active Project intervention will be delivered to intervention recipients via a face to face group exercise class by the Project Leaders (intervention providers). The intervention will be supported by ‘distance’ delivery at a population-level via the school’s digital media (social media), newsletter and posters displayed in the school. |
|  | **WHERE** |  |
| **7.** | Describe the type(s) of location(s) where the intervention occurred, including any necessary infrastructure or relevant features. | The Girls Active Project intervention will be delivered after school on school grounds (sports hall and playing field). |
|  | **WHEN and HOW MUCH** |  |
| **8.** | Describe the number of times the intervention was delivered and over what period of time including the number of sessions, their schedule, and their duration, intensity, or dose. | The Girls Active Project intervention will be delivered for 45-minutes once a week, after school. It will be delivered every Tuesday starting at 4pm, during the academic term, January to May 2021, excluding holidays. |
|  | **TAILORING** | N/A (intervention not yet delivered). |
| **9.** | If the intervention was planned to be personalised, titrated or adapted, then describe what, why, when, and how. |  |
|  | **MODIFICATIONS** |  |
| **10.^ǂ^** | If the intervention was modified during the course of the study, describe the changes (what, why, when, and how). | N/A (intervention not yet delivered). |
|  | **HOW WELL** |  |
| **11.** | Planned: If intervention adherence or fidelity was assessed, describe how and by whom, and if any strategies were used to maintain or improve fidelity, describe them. | Fidelity of intervention delivery will be assessed using the Project Leader logbooks.  Further details will be provided in the Girls Active Project feasibility study paper. |
| **12.^ǂ^** | Actual: If intervention adherence or fidelity was assessed, describe the extent to which the intervention was delivered as planned. | N/A (intervention not yet delivered). |

** **Authors** - use N/A if an item is not applicable for the intervention being described. **Reviewers** – use ‘?’ if information about the element is not reported/not sufficiently reported.

† If the information is not provided in the primary paper, give details of where this information is available. This may include locations such as a published protocol or other published papers (provide citation details) or a website (provide the URL).

ǂ If completing the TIDieR checklist for a protocol, these items are not relevant to the protocol and cannot be described until the study is complete.
* We strongly recommend using this checklist in conjunction with the TIDieR guide (see doi: <https://doi.org/10.1136/bmj.g1687>) which contains an explanation and elaboration for each item.

* The focus of TIDieR is on reporting details of the intervention elements (and where relevant, comparison elements) of a study. Other elements and methodological features of studies are covered by other reporting statements and checklists and have not been duplicated as part of the TIDieR checklist. When a **randomised trial** is being reported, the TIDieR checklist should be used in conjunction with the CONSORT statement (see [www.consort-statement.org](http://www.consort-statement.org)) as an extension of **Item 5 of the CONSORT 2010 Statement.** When a **clinical trial** **protocol** is being reported, the TIDieR checklist should be used in conjunction with the SPIRIT statement as an extension of **Item 11 of the SPIRIT 2013 Statement** (see [www.spirit-statement.org](http://www.spirit-statement.org)). For alternate study designs, TIDieR can be used in conjunction with the appropriate checklist for that study design (see [www.equator-network.org](http://www.equator-network.org)).
